# Antimicrobial Efficacy of Chemomechanical Carie Removal Agents—A Systematic Integrative Review

**DOI:** 10.3390/biomedicines12081735

**Published:** 2024-08-02

**Authors:** Adélaïde Janvier, Marie Maziere, Célia F. Rodrigues, Ana Paula Lobo, Paulo Rompante

**Affiliations:** 1 Dental Sciences, University Institute of Health Sciences (IUCS—CESPU), 4585-116 Gandra, Portugal; 2 UNIPRO—Oral Pathology and Rehabilitation Research Unit, University Institute of Health Sciences (IUCS—CESPU), 4585-116 Gandra, Portugal; 3 Associate Laboratory i4HB—Institute for Health and Bioeconomy, University Institute of Health Sciences—CESPU, Avenida Central de Gandra 1317, 4585-116 Gandra, Portugal; 4 UCIBIO—Applied Molecular Biosciences Unit, Translational Toxicology Research Laboratory, University Institute of Health Sciences (1H—TOXRUN, IUCS—CESPU), 4585-116 Porto, Portugal; 5 LEPABE—Laboratory for Process Engineering, Environment, Biotechnology and Energy, University of Porto, 4200-465 Porto, Portugal

**Keywords:** dental caries, minimal invasive, chemomechanical caries removal system, antimicrobial efficacy

## Abstract

Background: Dental caries is the most common oral disease in the world. When treatable, the drilling method continues to be used. This technique has its disadvantages because it is invasive and nonspecific. Chemomechanical carious tissue removal agents (CCRAs) such as Carisolv™ or Papacarie^®^ are non-invasive products that allow for the specific elimination of infected dentin. On the other hand, cariogenic bacteria are largely responsible for the initiation and development of lesions. Objectives: The aim is to analyze whether CCRAs have a relevant antimicrobial effect on cariogenic bacteria. Methods: A bibliographic search strategy was carried out in online databases using PRISMA 2020. The evaluation of the antibacterial efficacy of CCRAs was carried out through the analysis of the reduction in CFUs of cariogenic bacteria, and the presence of bacterial deposits, TVC, SVC and LVC by comparison with conventional drilling methods. Results: The results showed that the percentage of reduction in TVC, SVC and LVC for each agent is mostly high, but not significantly different from mechanical methods. The best results were found with CCRAs when compared to polymeric drills. The results also showed that there is a lack of methodological standardization. Conclusions: CCRAs have been shown to have a relevant antimicrobial effect on cariogenic bacteria; however, more studies need to be carried out using standardized methodologies.

## 1. Introduction

Dental caries is presently the most prevalent oral non-communicable disease in the world. It is a major public health problem, which has a daily impact on people’s well-being [1]. The multi-factorial etiology of caries is mainly associated with three factors: oral bacteria present in dental plaque, fermentation of carbohydrates and the presence of tooth surface [2]. There is a strong association between pH and the formation of dental caries, described by the Stephan curve [3]. The curve is divided into three parts: a rapid reduction in pH due to fermentation of carbohydrates by bacteria, followed by demineralization of enamel if the pH drops below 5.5 and a gradual increase back to baseline within 30 to 60 min. Tooth decay occurs when the pH remains below 5.5 for too long [3].

Traditionally, dental caries is treated using the conventional drilling method (CDM), i.e., excavating the carious tissue with traditional burs [4]. However, this technique has its drawbacks. The heat produced by the bur’s rotation can affect the pulp, causing its inflammation [5]. In addition, this technique can require local anesthesia when involving dentine tissues, which, combined with the noise of the burs, is often a source of anxiety, especially for children [6]. There is a tendency to over-prepare the cavities, eliminating both infected and affected dentine [7]. Moreover, there has been a paradigm shift in recent years regarding the treatment of dental caries, moving from “extension for prevention to the prevention of extension” [4].

Although the conventional technique is fully accepted by the scientific community, research on new therapeutic techniques is attempting to overcome its disadvantages [6]. The chemomechanical method has been developed to treat tooth decay for dentine lesions, particularly for children, anxious patients and patients that have a disability. Chemomechanical carie-removing agents (CCRAs) are non-invasive products that use a chemical compound to specifically eliminate the infected dentine [4,8]. They are classified into two categories: sodium hypochlorite (NaOCl)-based agents and enzyme-based agents [5]. The dentine softened by these products can then be removed using non-cutting tip instruments [6]. CCRAs allow for the elimination of infected dentin while preserving the affected dentin. Dentin is made up of 20% organic matrix, represented mainly by collagen fibers. During the caries process, the organic matrix is demineralized and the collagen fibers undergo structural changes. It is this degradation of collagen, or lack of it, that makes it possible to distinguish infected dentin from affected dentin. In affected dentin, the collagen fibers are intact and there is therefore the possibility of remineralization. There is therefore a critical point in preserving this layer of dentin, which is allowed by the use of CCRAs. They are very expensive products; so, they are not often used in the daily practice of dentistry. For example, for 3 mL of product, Papacarie costs approximately EUR 120, Brix3000 EUR 90 and Carisolv EUR 170. CCRAs have been emerging on the market since the 1970s [9]. In the 1970s, some scientists developed and tested GK 101, based on 5% sodium hypochlorite [10,11,12]. Later, they changed the glycine for amino butyric acid, resulting in a product sold under the name GK-101E or Caridex^®^. Still, these products were rarely used because they were time-consuming and aggressive to healthy oral tissues. In 1998, Carisolv^®^ was developed by MediTeam in Sweden. Papacarie^®^ was launched later, in 2003, in Brazil. It is a gel based on papain, chloramine and toluidine. In 2011, Carie-Care™ was developed in India, with a composition based on papain and essential oils from plant sources such as clove [13]. More recently, in 2016, Brix3000^®^, a gel in which papain is bio-encapsulated using EBE Technology (Encapsulating Buffer Emulsion), was developed in Argentina [9].

While there are many papers studying different CCRAs, few deal with their antimicrobial effect, which is necessary in the fight the underneath infectious disease. Additionally, there are few reports that exhaustively compare the different products. The aim of this study is therefore to evaluate the antimicrobial efficacy of CCRAs on the bacteria responsible for dental caries through a systematic review.

## 2. Methods

### 2.1. Bibliographic Research Method

This study was conducted in accordance with the Preferred Reporting Items for Systematic Reviews and Meta-Analyses (PRISMA), and this systematic review was registered on the international prospective register of systemic reviews (PROSPERO, id: CRD42024551489).

The research question was defined in accordance with the Population, Intervention, Comparation, Outcome (PICO) policy format (Table 1): Do CCRAs have a relevant antimicrobial effect on cariogenic bacteria?

A specific bibliographic search strategy was developed and implemented using the following online databases: PubMed, Science Direct, Web of Science (10 January 2024) with the keywords and Mesh terms linked to the review focus: “*microbiology*”, “*microbial culture*”, “*bacteria*”, “*microbiological assessment*”, “*anti-infective agents*”, “*chemomechanical*”, “*carie removal*” and “*chemomechanical caries removal agent*”. No time limit was defined (Table 2).

The inclusion criteria covered papers that assess the antibacterial effect through CFU reduction in cariogenic bacteria, presence of bacteria deposits, TVC, SVC, and LVC. The exclusion criteria included papers that did not assess the antimicrobial effect nor the effect on carious lesions. Papers that only assessed cytotoxicity on oral of mucosa cells, review paper, and book chapter were excluded too. Two independent reviewers were involved in the quality assessment process to minimize bias/errors. Any discrepancies in reviewers’ judgments were resolved through discussion and consensus. When consensus was not reached, a third reviewer arbitrated to achieve agreement.

### 2.2. Data Extraction

The data, namely, the variables to be analyzed, were extracted from in vivo studies in humans, in vitro in bacteria and fungi and ex vivo in extracted teeth. The analysis was performed by comparing the CCRAs, using as criteria the representativeness of the sample, the distribution of the groups, the existence of control groups, the sample collection procedures, the average exposure time, the caries’ excavation and the type of test carried out, the results of reduction in colony-forming units (CFUs) in percentage, the presence of bacterial deposits, the total viable count (TVC), the viable streptococcal count (SVC), and the viable lactobacillus count (LVC). The CFUs’ before and after contact with the CCRA or/and mechanical removal system was transformed into a percentage of reduction, in order to normalize the data and facilitate comparisons between CCRAs.

### 2.3. Data Processing

The percentage of reduction was calculated using values before and after caries excavation to normalize the results for comparison. For the paper published by Inamdar et al., the before and after values were defined by adding or removing the standard deviation, respectively [14]. Next, the following calculation was performed: (100 × after value)/before value. This provided the percentage of bacteria remaining in the cavity, which was subtracted from 100 in order to give the percentage reduction for each agent tested.

By collecting and processing the data in the papers and comparing them with so-called traditional methods, we were able to conduct a comprehensive analysis of the efficacy of CCRAs on cariogenic bacteria. When the CDM is compared to CCRAs, the first method is superior in reducing the number of bacteria present but when comparing with Pb, as a minimally invasive method, CCRAs have a greater antimicrobial efficacy.

To analyze the percentage of reduction, values before and after caries excavation were used in order to normalize the results for comparison. In the specific case of the results of Inamdar et al., the values before and after were defined by adding or removing the deviation standard, respectively [14]. A calculation was performed, [(100 × subsequent value)/previous value], in order to obtain the percentage of remaining bacteria and the percentage reduction for each agent tested.

By collecting and processing data from the articles and comparing them with so-called traditional methods, a comprehensive analysis of the effectiveness of CCRAs on cariogenic bacteria was carried out.

## 3. Results and Discussion

Based on our literature review, in which we selected 19 papers, we were able to draw up a specific analysis of the CCRAs present in the literature, their chemical composition and their efficacy against the bacteria responsible for dental caries.

### 3.1. Bibliographic Research

**Figure 1:**
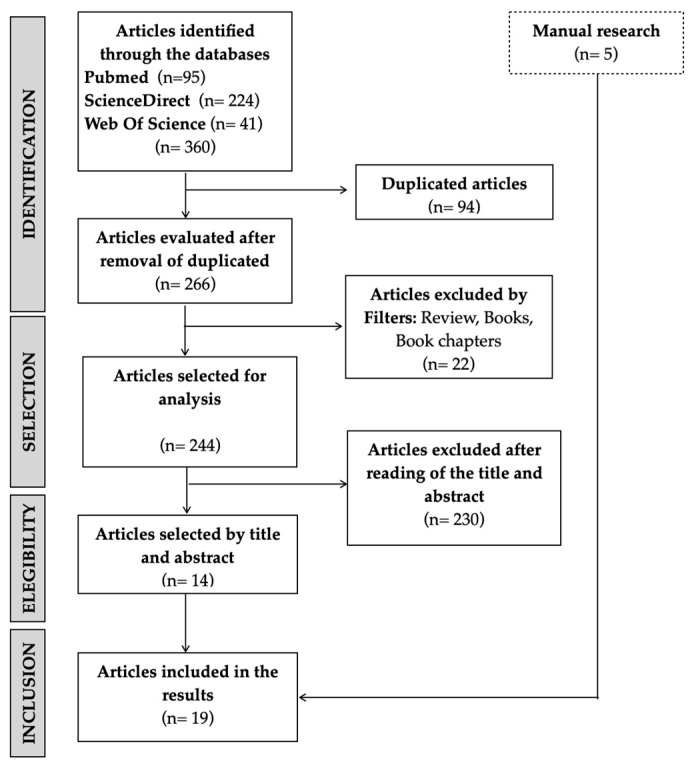
Flowchart.

With the advanced search, a total of 360 papers were found on the three different databases. Next, 94 papers were excluded for being duplicates and 22 according to the exclusion criteria. In the selection process (analysis of the title and abstract), 230 papers were excluded according to the eligibility criteria. A manual search allowed us to find five more papers to include in this review. A total of 19 papers were included (Figure 1).

### 3.2. Chemomechanical Carie Removal Systems (CCRA)

The scientific literature focused more on Papacarie^®^ and Cariesolv™ than on other systems. Papain is the main active compound in all CCRAs, except for Cariesolv™, which contains sodium hypochlorite (NaOCl) 0.5%, and three amino acids: glutamic acid, leucine and lysine (Table 3).

CCRAs allow for the elimination of the infected dentine while preserving the affected one [4,8]. The antimicrobial mechanisms are based on two active agents: NaOCl and papain.

All sodium-hypochlorite-based gels work in the same way. Carisolv™ is the most advanced version, containing three amino acids, namely, lysine, leucine and glutamic acid, which replaced aminobutyric acid to neutralize the aggressive effect of sodium hypochlorite on healthy oral tissues [13]. Even though the antibacterial efficacy of NaOCl is recognized, the exact mechanism of antimicrobial efficacy is not well elucidated. It is mainly attributed to its constituent free available chlorine, comprising hypochlorite (OCl^–^) and hypochlorous acid (HOCl), both potent oxidizers that act by dissolving and disrupting the structure of the biofilm [32]. Enzyme-based CCRAs are mainly composed of papain, an endoprotein derived from the adult green papaya, *Carica papaya*. It resembles human pepsin, with bactericidal, bacteriostatic and anti-inflammatory activity [8]. Papain acts in several ways: it disrupts the membrane permeability of bacteria by breaking the amino acid bonds of Gram-negative bacteria. Its bactericidal and bacteriostatic effects inhibit Gram-positive and Gram-negative bacteria [30]. The antibacterial activity of papain is not due only to its proteolytic activity, but also to other enzymatic actions, such as amidase and esterase activities [33]. Additionally, papain also digests dead cells [8]. Papacarie^®^ is the most studied in scientific articles. It contains a small amount of chloramine and toluidine blue, a dye that acts against *Streptococcus mutans* [8]. Brix 3000^®^ is the most recent and innovative agent. The papain is encapsulated, enabling the enzymatic activity to be concentrated at 3000 U/mg. Encapsulation of the papain allows for immobilization and therefore confers stability to the endoprotein, which will exponentially increase the proteolytic activity of the product, reducing the risk of dissolution by oral fluids and allowing for better storage as there is no longer any need to respect the cold chain preservation [9].

### 3.3. Antibacterial Effect of CCRA

To assess the antimicrobial efficacy of CCRAs, the reduction in bacteria after caries excavation needs to be analyzed and equated. This leads to a major problem, which is defining what caries-free is and the quantity of bacteria that can remain in the cavity without risking disease progression [34]. Calculating the number of bacteria acceptable after caries excavation is a difficult task, as it can be influenced by many things, such as the sample collection procedure [35] or the unreliability of visual and tactile techniques such as caries detectors [36]. Kidd et al. stated that values below 10^2^ are acceptable ones for *Lactobacillus* spp. and *S. mutans* after the conventional drilling method (CDM) [37]. However, more recent studies have shown, owing to the use of the PCR method, that traditional bacterial culture methods underestimated the true number of bacteria remaining in a caries-free cavity [34]. It is currently estimated that a number of microorganisms between 10^1^ and 10^4^ CFUs/mL remain clinically sound in the dentine [36].

**Figure 2:**
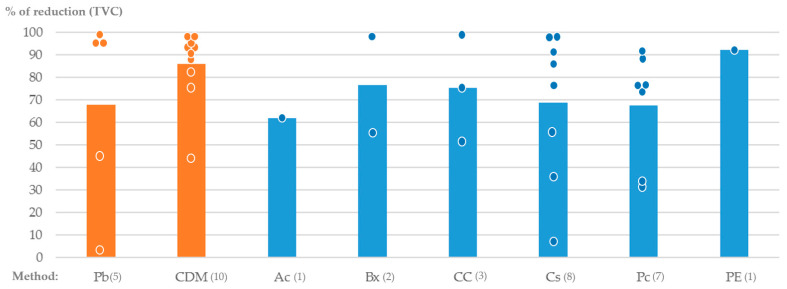
Percentage of reduction in total viable count (%) for each method tested. Each point represent the exact value found in studies.

The total viable count (TVC) is expressed in colony-forming units per milliliter (CFUs/mL), and estimates the number of microorganisms present in a sample. In the papers considered in this review, all microorganisms present in the sample were included; therefore, no distinction was made between the oral cariogenic bacteria in the TVC of the papers studied in this review. The main agents of caries are *Streptococci* and *Lactobacilli* [2]. *S. mutans* is the organism that plays a significant role in dental caries initiation, while *Lactobacillus* spp. is the organism with the greatest influence on its progression [38]. Both cariogenic bacteria produce lactic acid during lactic fermentation, which is necessary for their survival. In contact with this acid, the enamel undergoes a solubilization process known as demineralization [2]. The percentage of reduction in TVC found for each agent is, for the great majority, high but not significantly different from that observed with mechanical methods (Table 4 and Figure 2).

As for the TVC, the reduction in the *Streptococcus* spp. viable count (Table 5) and the *Lactobacillus* spp. viable count were calculated, using the same strategy as explained above (Table 6).

Carisolv™ was shown to be the best method for bacterial reduction in *Streptococcus* spp. [19] and PapEdent was the best one for bacterial reduction in *Lactobacillus* spp. [28]. Nevertheless, for both *Streptococcus* spp. and *Lactobacillus* spp. viable counts, the percentage of reduction in all the agents (Brix 3000^®^, Carie-Care™, Carisolv™, Papacarie^®^, PapEdent) was above 80% [17,19,24,25,28,31], except for Papacarie^®^ in one paper where the *Lactobacillus* spp. viable count was below 70% [25].

In general, when the conventional drilling method was compared to CCRAs (Table 7), the first was superior to the latter in reducing the number of bacteria present. The conventional method showed a significantly greater reduction in TVC than Carisolv™. Consequently, the conventional method has a greater antimicrobial efficacy than CCRAs [15]. The same results were found in other papers, although none of the results were statistically significant [18,20,21,22,26,30]. Moreover, the antimicrobial efficacy of CCRAs was present, yet less powerful than that of CDM. The fact that there was no significant difference between CCRAs and CDM clearly shows that both are effective in reducing the number of bacteria present in a cavity. Only one paper found that Papacarie^®^ had no antimicrobial activity [27]. Papacarie^®^ and Brix 3000^®^ were shown to have a greater percentage of bacterial reduction than CDM, although the results were not statistically significant [9,23,24,31]. We can explain these results by a simple fact: in CDM, more tissue is eliminated and there is a proportionally greater chance of reducing the number of bacteria, whereas with CCRAs, the objective is to only remove the infected dentine. There is a tendency to over-prepare cavities with CDM since there is no associated tactile sensation and traditional burs do not differentiate between infected and affected dentine [20]. However, this is not necessarily useful as long as the threshold of 1 × 10^4^ CFUs/mL, currently recognized as acceptable, is not exceeded [37]. This was the case for most studies which met our eligibility criteria [15,16,18,22,26,27,30].

Polymer burs (Pb) are used as minimally invasive mechanical techniques. They are made from a polymer whose hardness is greater than that of the infected dentine, but less than that of the affected dentine, enabling the former to be specifically removed [20]. The majority of articles comparing the two (Table 7) show that CCRAs have a greater antimicrobial effect than Pb, although the results are not significant for some papers [14,18,21]. Carisolv™ reduced CFUs significantly better than Pb [20]. Only one paper found that Pb had a greater reduction than Carie-Care™, even though the difference was very small [29].

### 3.4. Clinical Implications

CCRAs represent a very good alternative from a microbiological point of view and with a view to carry out minimally invasive treatment. In fact, they make it possible to treat a carious lesion in dentine without recourse to anesthesia while enabling infected dentine to be specifically removed. From a clinical point of view, this is a not-inconsiderable detail in the context of a pedodontics treatment. When compared with Pb, bacterial reduction results are better for CCRAs. CCRAs should therefore be considered an option in their own right for the treatment of dental cavities.

### 3.5. Methodological Bias and Limitations

The lack of standardization of studies is evident. In general, all articles follow a precise protocol to determine the antimicrobial effect of the CCRA studied; however, there are notable differences in the protocol steps (Table 8). Firstly, most authors chose to exclude uncooperative patients [9,14,17,20,21,23,29,31], which can influence the results obtained when compared with those who have kept them. In some studies where the research was carried out in vivo, no rubber dam isolation was achieved, only relative isolation [9,23]. Regarding the teeth samples, with the exception of one study (which used bovine teeth), the teeth were human [27]. Some studies chose to work on deciduous teeth [17,18,19,20,21,23,24,26,28,29,31], while others worked on permanent teeth [9,14,15,16,22,25,30]; however, there were significant differences between the two, namely, in the enamel and dentine mineralization [39]. Most of the studies dealt with teeth that were already decayed [9,14,15,17,18,19,20,21,22,23,24,26,28,29,30,31] but some protocols resorted to a bacterial contamination protocol to mimic tooth decay [16,25,27]. Importantly, the CCRAs’ operating time on the tooth did not always follow the manufacturer’s recommendations, which may also influence and introduce bias into the results [16]. In regard to the media used to grow the dentin samples, several different media were also applied (e.g., Schaedler agar, Mitis Salivarius agar [17,28], MacConkey agar [30] or Blood agar [9,14,23,24]), and similarly, the methods of bacterial contamination wass not the same for all studies. While the majority used bacterial culture [9,14,17,18,19,20,21,23,24,25,27,28,29,30,31], some studies used microscopy and others did not [15,18,22,26]. It is, however, relevant to note that the microscope does not provide an overall view as it only shows a slice of the whole, which can condition the bacterial numbers. The count method was carried out manually in some studies [24] and colony counters were used in others [14,17,23,27,28], but most studies do not mention how the count was performed [9,15,16,18,19,20,21,22,25,26,29,30,31]. In addition, the subjectivity of the visual and tactile techniques used in studies to assess whether caries excavation was complete or not can result in variability [26]. All these methodological particularities arose and are classified as limitations with the capacity to interfere with the results of the studies and, therefore, to be interpreted as generating bias.

Even though the papers showed the antimicrobial effect of CCRAs, more studies must be conducted to assess the acceptable number of bacteria that can remain in a caries-free cavity to fix the relevancy of this effect. In this sense, recent studies were carried out to try and potentiate the antimicrobial effect of CCRAs, mainly the Papacarie^®^, by adding methylene blue to its composition, resulting in a product called PapaMBlue [40]. Blue methylene, which is a photosensitizer, was shown to be activated by light in photodynamic therapy, an adjunctive therapy to the treatment of carious lesions. The combination of the two seems to increase the antimicrobial effect of CCRA. Although other photosensitizers such as *Bixa orellana* extract have been studied [41], there is little research on this promising and innovative subject.

## 4. Conclusions

CCRAs have been shown to have a relevant antimicrobial effect on cariogenic bacteria; however, more studies need to be carried out using standardized methodologies.

Both sodium hypochlorite and enzyme-based CCRAs show an important bacterial reduction in the TVC, SVC and LVC.

The Brix 3000^®^, is the CCRA with the better results, followed by the Papacarie^®^. Enzyme-based CCRAs can then be assumed to be the best ones for reducing cariogenic bacteria, even though the conventional drilling method has better results. However, when compared to another minimally invasive technique such as polymer bur, all CCRAs show superior results, which makes them leaders in this category. Nevertheless, the key absence of standardization among the papers exposes the need of further studies to put CCRAs among the most widely used caries excavation techniques.

More studies need to be carried out using standardized methodologies.

## Figures and Tables

**Table 1:** Focused research questions presented using the PICO.

Element	Description
Population	Cariogenic bacteria
Intervention	Chemomechanical removal of decayed dentin
Comparison	Compare the different CCRAs
Outcome	Antimicrobial efficacy

**Table 2:** Search strategy.

Database	Advanced Search
PubMed	(“microbiology” [MESHTerms] OR microbial culture OR “bacteria” [MeSH Terms] OR microbiological assessment) AND (“anti-infective agents” [MeSH Terms]) AND ((Chemomechanical) AND (carie removal)) OR (chemomechanical caries removal agent)
Science Direct	(microbiology OR microbial culture OR bacteria OR microbiological assessment) AND (anti-infective agents) AND ((Chemomechanical) AND (carie removal)) OR (chemomechanical caries removal agent)
Web of Science	(microbiology OR microbial culture OR bacteria OR microbiological assessment) AND (anti-infective agents) AND ((Chemomechanical) AND (carie removal)) OR (chemomechanical caries removal agent)

**Table 3:** CCRAs and their respective composition.

Name	Creation	Manufacturer	Active Agent	Excipient	Paper
**Cariesolv ™**	1998	Maditeam, **Sweden**	NaOCl 0.5%, + Amino Acid (glutamic acid, leucine, lysine)	Carboxymethylcelulose, sodium chloride, sodium hydroxide	[15,16,17,18,19,20,21]
**Papacarie^®^**	2003	PulpDent, **Brasil**	Papain	Chloramine, Toluidine blue	[16,17,18,22,23,24,25,26,27]
**PapEdent^®^**	2008	SubramaniamP. and Gilhotra K., **India**	Papain, D-α-tocopherol acetate	Distilled Water, glycerine, amylopectin, carbopol, propyl-p-hydroxybenzoate, coloring agent (green apple)	[28]
**Carie-Care™**	2011	Uni-biotech Pharmaceuticals PrivateLimited, **India**	Papaine, therapeutic Essential Oils	Coloring Gel (Blue), Sterile water, Chloramine & Sodium Chloride, Permitted Color (Blue), Sodium Methyl Paraben & Sodium Propyl Paraben.	[14,19,29]
**Apacaries gel^®^**	2012	Apa Juntavee, **Thailand**	Papain, Polyphenols (*Mangosteen* extracts)	n/a	[30]
**Brix3000^®^**	2016	Brix SRL, **Argentina**	Bioencapsulation of Papain (3000 U/mg)	Propylene Glycol, Citric Pectin, Triethanolamine, Sorbitan Monolaurate, Disodium Phosphate, Monopotasic Phosphate, Toluidine Blue, Distilled Water	[9,14,31]

**Table 4:** Percentage of reduction in total viable count (%).

Chemomechanical Methods						Mechanical Methods		Papers
Ac	Bx	CC	Cs	Pc	PE	Pb	CDM	
			35.71				92.86	[15]
				73.3			93.3	[22]
					92.36			[28]
			86.17				44.35	[23]
			7.8	31.9				[16]
				88.47			88.29	[24]
			92.02	92.25				[17]
			56.7	76.7		3.3	90	[18]
		75.97	76.87					[19]
		99.9				99.9		[29]
				76.7			83.30	[26]
	98.63						95.40	[9]
	54.73	50.40				46.66		[14]
61.86							75.93	[30]
			97.59			95.04	98.14	[20]
				33.3				[27]
			97.58			95.03	98.13	[21]

Ac: Apacaries gel; Bx: Brix3000; CC: Carie-Care^®^; Cs: Cariesolv ™; CDM: Conventional drilling method; Pb: Polymer burs; Pc: Papacarie^®^; PE: PapEdent.

**Table 5:** Percentage of reduction in *Streptococcus* spp. viable count (%).

Chemomechanical Methods				Mechanical Method	Paper
Bx	Cs	CC	Pc	CDM	
	90.26	88.54			[19]
			80.83		[25]
87.46				86.40	[31]

Bx: Brix3000; CC: Carie-Care™; Cs: Cariesolv ™; CDM: Conventional drilling method; Pc: Papacarie^®^.

**Table 6:** Percentage of reduction in *Lactobacillus* spp. viable count (%).

Chemomechanical Methods					Mechanical Method	Paper
Bx	CC	Cs	Pc	PE	CDM	
				94.10		[28]
			83.36		81.05	[24]
		86.93	87.36			[17]
	83.2	83.2				[19]
			67.5			[25]
85.84					85.28	[31]

Bx: Brix3000; CC: Carie-Care™; Cs: Cariesolv™; CDM: Conventional drilling method; Pc: Papacarie^®^; PE: PapEdent.

**Table 7:** Comparison between chemo-mechanical methods and mechanical methods (%).

Ac-CDM	Bx-CDM	Cs-CDM	Pc-CDM	Bx-Pb	Cs-Pb	CC-Pb	Pc-Pb	Paper
		−57.15						[15]
			−20.00					[22]
			41.82					[23]
			0.17					[24]
		−33.3	−13.3		53.4		73.4	[18]
						−0.002		[29]
	3.23							[9]
			−6.6					[26]
				8.07		3.74		[14]
−14.07								[30]
		−0.55			2.55			[20]
		−0.55			2.55			[21]
	1.06							[31]

The red digits indicate the superiority of the CCRA method over mechanical methods. The green digits indicate the superiority of the mechanical method over the CCRA methods; Ac: Apacaries gel; Bx: Brix3000; CC: Carie-Care™; Cs: Cariesolv™; CDM: Conventional drilling method; Pb: Polymer burs; Pc: Papacarie^®^.

**Table 8:** Study designs’ description: a source of results’ variability.

Type of Study	Methods Tested	Sample/Specimen					Chemomechanical Methods			Mechanical Methods	Paper
		N	Type	Origin	Caries Lesions	Preparation	Time of Application (s)	Repetitions	Total Time (s)	Total Time (s)	
**Ex vivo**	Cs/CDM	14	M	H	Dentine caries	Separated in half	Cs: 30’	+	272’	CDM: 116’	[15]
	Pc/CDM	20	M	H	Dentine caries	Separated in half	Pc: 30’	+	328’	CDM: 125’	[22]
	Cs/Pc/CHX	30	PM	H	-	Section 2 mm dentin discs contaminated with SM	Cs, Pc, C: 300’	-	300’	-	[16]
	Pc/Cs/Pb/CDM	60	m	H	Moderated stages of caries lesion	Separated in half	Pc, Cs: 30’	+	Pc: 359.60′/Cs: 461.60′	CDM: 151′/Pb: 345′	[18]
	Cs/CC	40	m	H	Dentine caries	Separated in half	?	?	?	?	[19]
	Pc/C	20	M	H	-	Contaminated with SM and *L. casei*	Pc: 30’	3x	?	-	[25]
	Pc/CDM	30	m	H	Occlusal dentine caries	Separated in half	Pc: 30’-40’	+	351.56’	CDM: 158.41’	[26]
	Pc/CHX/PBS	60	I	B	-	Contaminated with SM and *L. casei*	Pc: 30’/CHX: 120’/PBS: 60’	?	?	-	[27]
**In vivo**	PE	20	m	H	Occlusal dentine caries	-	PE: 40’	+	?	-	[28]
	Pc/CDM	40	m	H	Occlusal dentine caries	-	Pc: 30’-40’	+	?	?	[23]
	Pc/CDM	50	m	H	Occlusal dentine caries and cervical lesion	-	Pc: 30’-40’	+	461’	CDM: 459’	[24]
	Pc/Cs	40	m	H	Large occlusal cavities	-	Pc: 60’/Cs: 30’	+	?	-	[17]
	CC/Pb	50	m	H	Occlusal dentine active caries	-	CC: 30-60’	+	?	?	[29]
	Bx/Pb	60	M	H	Occlusal dentine caries	-	Bx: 120′	+	2287′	Pb:1 433′	[9]
	Bx/CC/Pb	45	M	H	Dentine caries	-	Bx, CC: 180’	+	Bx: 819.6′/CC: ?	Pb: 1236′	[14]
	Ac/CDM	40	M	H	Occlusal dentine caries	-	Ac: 30’-40’	+	278.4′	CDM: 91.2′	[30]
	Cs/Pb/CDM	60	m	H	Occlusal dentine caries	-	Cs: 30’	+	455.45′	CDM: 113.25′/Pb: 129.20′	[20]
	Cs/Pb/CDM	20	m	H	Occlusal dentine caries	-	Cs: 30’	?	129.21′	CDM: 113.26′/Pb: 455.46′	[21]
	Bx/CDM	60	m	H	Occlusal dentine caries	-	Bx: 120′	+	1823′	CDM: 1200′	[31]

+: Yes, −: No, ?: not mentioned. Ac: Apacaries gel; B: Bovine; Bx: Brix3000; CC: Carie-Care™; CHX: Chlorhexidine Digluconate; Cs: Cariesolv™; CDM: Conventional drilling method; I: incisor; H: Human; M: Permanent molars; m: Decideous molar; Pb: Polymer burs; Pc: Papacarie^®^; PE: PapEdent; SM: *Streptococcus mutans*.
